# Antibiotic resistance in urinary tract infections: A study on trends and contributing factors in outpatient care among Indian patients

**DOI:** 10.6026/9732063002001908

**Published:** 2024-12-31

**Authors:** Ramya Sampathkumar, Saranya R., Deepika Gnanasekaran, Vanshikaa Thakran, Haroon Aslam, Shoraf Pascal

**Affiliations:** 1 Department of Internal Medicine, Blackpool Victoria Teaching Hospital, Blackpool, United Kingdom; 2 Department of Community Medicine, Madha Medical College and Research Institute, Chennai, Tamil Nadu, India; 3 Department of Internal Medicine, Hillingdon Hospital, London, United Kingdom; 4 Medical officer, Promestha Clinic, Gurugram, Haryana, India; 5 General Practitioner, Kasturba Medical College, Mangalore, Karnataka, India; 6 Department of Community Medicine, Madha Medical College and Research Institute, Chennai, Tamil Nadu, India

**Keywords:** Urinary tract infections, antimicrobial resistance, outpatient, escherichia coli, antibiotic stewardship, extended-spectrum β-lactamase

## Abstract

UTIs are quite a common infection in outpatient care; however, the rise of antimicrobial resistance raises considerable challenge. This study determines the trend of resistance among UTI pathogens and considers factors contributing to it, such as prescribing, which often occurs in an outpatient setting. It was a single-setting retrospective analysis of 80 outpatient UTI cases. This involved bacterial isolation, antimicrobial susceptibility testing and analysis of potential factors that may have led to resistance, such as antibiotic prescribing and patient comorbidities. Descriptive statistics were therefore applied in SPSS for data analysis. The most common pathogen was Escherichia coli (70%) and exhibited significant resistance to trimethoprim-sulfamethoxazole at 30% and to fluoroquinolones at 22%. Extended-spectrum β-lactamase (ESBL)-producing strains comprised 8% of *E. coli* isolates. Higher resistance rates were associated with inappropriate antibiotic use (p = 0.001), frequent use of antibiotics (p = 0.004) and comorbid conditions such as diabetes (p = 0.002). The levels of resistance to antimicrobials in outpatient UTIs are rising, especially due to the inappropriate prescribing and health conditions. Improvement of stewardship of antibiotics and accuracy of diagnosis are required in controlling trends in resistance seen in outpatient care.

## Background:

The most common bacterial infections seen in the ambulatory practice are urinary tract infections or UTIs among women, elderly patients and patients with chronic illnesses, like diabetes mellitus or immunosuppression [1]. UTIs contribute to a considerable proportion of ambulatory antibiotic prescriptions and thus represent a critical point of entry in the fight for effective antimicrobial stewardship [2]. Management of UTIs has become increasingly complex over the years due to the increasing number of uropathogens resistant to antibiotics, especially Escherichia coli, the cause of about 70-90% of uncomplicated UTIs, continues to increase [3]. Overuse and misuse of antibiotics have invariably increased resistance factors, especially in outpatient settings. Consequently, drug resistance has become alarmingly widespread in several regions, with resistance to commonly used antibiotics, such as trimethoprim-sulfamethoxazole and fluoroquinolones, standing at disturbingly elevated levels, thereby rendering treatment more failures, infections more complicated and recurrent [4, 5]. For example, resistance to trimethoprim-sulfamethoxazole, which is the first choice of treatment for uncomplicated UTIs, has exceeded 30% in certain areas. Therefore, alternative treatments are applied in such cases. Fluroquinolone resistance, which had been exceedingly rare in ambulatory settings, has become up to 20-30% of cases, barring additional treatment option [6]. Most current issues affecting treatment include the appearance of extended-spectrum β-lactamase (ESBL)-producing strains of *E. coli* and other uropathogens in the community. Such multi-resistant bugs are resistant to a wide range of β-lactam antibiotics, including penicillins and cephalosporins, which have long been standard anti-UTI agents [7]. The patients infected with ESBL-producing strains generally tend to require stronger antibiotics, like carbapenems; this is a type of antibiotic not usually used for outpatient care and more expensive and associated with greater adverse effects [8]. Other inappropriate prescribing practices in outpatient care involve empirical prescriptions without confirmation of bacterial infection and abuse of broad-spectrum antibiotics, thereby playing a considerable role in developing resistance [9]. Patients suffering from recurrent UTIs with frequent exposure to antibiotics or those with underlying conditions like diabetes are more likely to develop resistant infections [10]. Therefore, it is of interest to monitor and examine the antimicrobial resistance trends of UTI-causing pathogens in outpatient settings and later establish determinants such as prescription patterns and comorbidities among the patients. Understanding these factors may enable health care providers to improve upon the management of UTI and regulate antibiotic use that would then control the rise of resistance.

## Methodology:

This retrospective study was conducted on 80 patients who were diagnosed with urinary tract infections in an outpatient clinic from January 2022 to December 2023. It addressed the issues related to antimicrobial resistance patterns and the factors responsible for such resistance.

## Inclusion criteria:

[1] Adult patients aged 18 years and above.

[2] Positive urine cultures confirming bacterial UTI.

## Exclusion criteria:

[1] Patients with complicated UTIs (*e.g.*, catheter-associated infections).

[2] Patients hospitalized within the last 30 days.

## Data collection:

## Bacterial isolates and susceptibility testing:

Urine samples were cultured and antimicrobial susceptibility testing was performed using the disk diffusion method. Results were interpreted according to Clinical and Laboratory Standards Institute (CLSI) guidelines.

## Antibiotic resistance patterns:

The resistance patterns of the most common UTI pathogens to antibiotics, including trimethoprim-sulfamethoxazole, fluoroquinolones, cephalosporins and nitro-furantoin, were recorded.

## Contributing factors:

Data on patient demographics (age, gender), comorbidities (*e.g.*, diabetes) and antibiotic prescription history (frequency and type) were collected from medical records. Prescribing practices were evaluated for appropriateness based on clinical guidelines.

## Statistical analysis:

Statistical analysis was performed using SPSS software (version 26). Continuous variables were expressed as mean ± standard deviation (SD) and categorical variables as percentages. Logistic regression was used to identify factors contributing to antimicrobial resistance. A p-value of <0.05 was considered statistically significant.

## Results:

A total of 80 patients with culture-confirmed urinary tract infections were included in the study (Table 1). The distribution of bacterial pathogens and their resistance patterns to commonly prescribed antibiotics are presented in Table 2. Most patients were female, with a considerable proportion having diabetes or a history of recurrent UTIs, both of which are known risk factors for resistant infections (Table 3). *E. coli* was the most isolated pathogen, accounting for 70% of UTI cases in this outpatient cohort (Table 4). High resistance rates to trimethoprim-sulfamethoxazole and fluoroquinolones suggest the need for alternative empiric therapies in this population. 8% of *E. coli* isolates were ESBL producers, indicating a significant presence of multidrug-resistant strains (Table 5). Patients who had been prescribed three or more courses of antibiotics had a significantly higher rate of resistant infections (Table 6). The presence of diabetes was significantly associated with higher rates of antibiotic resistance. Longer durations of antibiotic therapy were linked to higher resistance rates, underscoring the importance of appropriate duration of treatment (Table 7). Empirical prescribing without culture testing was associated with a higher rate of resistant infections, highlighting the need for more targeted therapy based on culture results (Table 8). Overall resistance rate was significantly higher with broad-spectrum antibiotics compared to first-line antibiotics; however, the message is to avoid overuse of broad-spectrum agents (Table 9). Recurrent UTI history patients were at significantly elevated risk to harbour resistant infections, likely from antibiotic exposure events in multiple counts (Table 10).

## Discussion:

The results of the study underscore the rising burden of outpatient urinary tract infection-related antimicrobial resistance, especially in E. The most prevalent species of *E. coli* that was recovered constituted 70% of all infections analyzed [11]. Of major concern is the high resistance rates to antibiotics prescribed commonly, like trimethoprim-sulfamethoxazole at 30% and fluoroquinolones at 22%, which is a resistance rate that mirrors the global trend in antimicrobial resistance [12]. These findings therefore mean empiric treatment guidelines for UTIs need to be reassessed, particularly in regions where resistance rates exceed those at which a given antibiotic still is effective [13]. The other complicating factor includes the presence of extended-spectrum β-lactamase (ESBL)-producing *E. coli* strains, amounting to 8% of the isolates. ESBL production gives the organism resistant to a vast number of β-lactam antibiotics, such as penicillins and cephalosporins that are used frequently in the outpatient management of UTIs [14]. Patients infected with ESBL-producing strains should be treated with either carbapenems or other advanced antibiotics, which are not favoured for ambulatory administration because of high expense and possible side effects [15]. The exposure of these multidrug-resistant organisms to ambulatory settings calls for added vigilance and tightening up on the stewardship programmes of antibiotics [16]. Antibiotic inappropriateness was a significant attribute that led to resistance in this study, particularly through empirical prescribing without urine cultures for confirmation. In empirical therapy, resistance levels stood at a much higher rate than in those whose treatment was culture-based, at 45 percent of cases [17]. The results, therefore, support the call for increased application of diagnostic testing, like urine cultures, in outpatient care before prescription with antibiotics [18]. Additionally, the overuse of broad-spectrum antibiotics, such as fluoroquinolones, was associated with increased rates of resistance and this again brought to the forefront the importance of reserving these agents for situations in which first-line treatments have failed [19]. The presence of comorbid conditions was significantly associated with the development of resistant infections, with 37% of diabetic patients showing antibiotic resistance compared with 19% of their non-diabetic counterparts. Diabetes is an established risk factor for recurrent UTIs and antibiotic resistance due to its association with compromised immune function and frequent health care encounters [20]. These findings show that special concern should be placed on diabetic patients with UTIs, as antibiotic sensitivity should be assessed carefully and treatment efficacy closely monitored [21]. Duration of antibiotic therapy was also a key factor; courses longer than 7 days in duration are associated with higher rates of resistance. This again underlines the need to follow clinical guidelines relating to the shortest effective course of antibiotics to avoid risk of developing resistance [22]. Those patients with a history of recurrent UTIs were at significantly increased risk to have resistant infections; most probably due to repeated exposure to antibiotics over time [23]. Antibiotic treatment for UTI is often empiric without urine culture or susceptibility testing and is usually based on national guidelines and local resistance profiles [24]. Most antimicrobials used to treat urinary tract infections, including SXT, can achieve high urinary concentrations [25].

## Conclusion:

Urinary tract infections in the outpatient setting are increasingly challenging due to the inappropriate use of antibiotics, comorbid conditions and the presence of organisms that are multidrug-resistant, like ESBL-producing *E. coli*. As such infections will be treated, there is a need for better diagnostic practices using urine cultures to ensure that the prescriptions are evidence-based and adopt judicious use of broad-spectrum drugs as a last resort only. A better approach to reduce the trend of resistance and preserve the effectiveness of current treatments for UTIs would be through strengthened antibiotic stewardship programs and education of patients about proper use of antibiotics.

## Figures and Tables

**Table 1:** Demographic characteristics of patients

**Variable**	**Number of Patients (%)**
Age (Mean ± SD)	52.3 ± 10.5
Gender (Female)	64 (80%)
Gender (Male)	16 (20%)
Diabetes	22 (27.5%)
Recurrent UTI History	18 (22.5%)

**Table 2:** Distribution of bacterial pathogens in UTI isolates

**Pathogen**	**Percentage of Isolates (%)**
Escherichia coli	70%
Klebsiella pneumoniae	15%
Proteus mirabilis	5%
Other pathogens	10%

**Table 3:** Antibiotic resistance patterns in *E. coli* isolates

**Antibiotic**	**Resistance Rate (%)**
Trimethoprim-sulfamethoxazole	30%
Ciprofloxacin (Fluoroquinolone)	22%
Amoxicillin-clavulanate	15%
Cephalexin	12%
Nitrofurantoin	4%

**Table 4:** Prevalence of ESBL-producing *E. coli*

**ESBL-Producing *E. coli***	**Percentage of Isolates (%)**
Yes	8%
No	92%

**Table 5:** Frequency of antibiotic use and resistance rates

**Number of Antibiotic Courses**	**Resistance Rate (%)**	**p-value**
1-2	18%	
3 or more	34%	0.003

**Table 6:** Impact of comorbidities on resistance

**Comorbidity**	**Resistance Rate (%)**	**p-value**
Diabetes	37%	0.002
No comorbidities	19%	

**Table 7:** Duration of antibiotic therapy and resistance rates

**Duration of Therapy (Days)**	**Resistance Rate (%)**	**p-value**
Less than 7 days	17%	
More than 7 days	28%	0.021

**Table 8:** Prescribing practices and appropriateness

**Prescribing Practice**	**Percentage of Cases (%)**	**p-value**
Empirical without testing	45%	
Culture-based	55%	0.004

**Table 9:** Comparison of first-line and broad-spectrum antibiotic use

**Antibiotic Type**	**Resistance Rate (%)**	**p-value**
First-line (*e.g.,* TMP-SMX)	25%	
Broad-spectrum (*e.g.,* fluoroquinolones)	35%	0.015

**Table 10:** Correlation between recurrent utis and resistance

**Recurrent UTI History**	**Resistance Rate (%)**	**p-value**
Yes	36%	0.001
No	21%	
